# From the black Atlantic to the bleak Pacific: Re-reading “Benito Cereno”

**DOI:** 10.1080/14788810.2017.1384612

**Published:** 2018-03-13

**Authors:** Alexandra Ganser

**Affiliations:** Department of English, University of Vienna, Vienna, Austria

**Keywords:** Archipelagic studies, “Benito Cereno”, black Atlantic, black Pacific, Herman Melville, piracy, slavery, transatlantic studies, transpacific studies

## Abstract

Herman Melville’s novella “Benito Cereno” (1855/56) is one of the best-studied texts both within Melville’s oeuvre and nineteenth-century American literature in general. In recent decades, its puzzling structure and fragmented narrative perspective as well as its symbolism and themes have been subject to critical scrutiny mostly in the context of inquiries into the text’s racial politics regarding the institution of slavery. More specifically, the canonical tale about a slave uprising on the ship *San Dominick*, its detection by a Massachusetts-born captain and its consequences, has been discussed in the context of Paul Gilroy’s black Atlantic paradigm. Few readings of the tale consider the significance of the Pacific setting of a story grounded in the transatlantic slave trade but happening far away from the center of American slavery. Taking a fresh look at Melville’s tale, this essay focuses on its translation of (black) Atlantic subject matters and epistemologies onto the Pacific. Not only do I read the tale as both an Atlantic and a Pacific text, demonstrating that the institution of slavery and its specters know no geographical borders in Melville’s imagination; I also argue that piracy is an important trope in this context. Anticipating the shift of piracy cases and slavery to the Pacific towards the end of the nineteenth century, it both recalls a black Atlantic and predicts a bleak Pacific of violent imperial scenarios that would come to characterize US–Pacific relations.

## Introduction

Critical readings of Herman Melville’s novella “Benito Cereno” (1855/56) rarely consider the significance of the Pacific island and coastal settings of a story grounded in the transatlantic slave trade. While Melville’s canonical tale about a slave uprising on the ship *San Dominick*, and its detection and suppression by an American captain has been insightfully discussed in the context of Paul Gilroy’s black Atlantic paradigm and New World slavery,^1^ the tale, in fact, starts off the coast of Chile, near the island of Santa María, and ends in Lima, the capital city of the Viceroyalty of Peru, where the rebellious slaves are tried and executed (with Babo’s head speared on the central Plaza) and where Cereno dies.^2^ My critique focuses on the significance of the novella’s Pacific contexts and subtexts, which provide an important literary commentary on American imperialist endeavors in the second half of the nineteenth century.

I read the tale as a transoceanic text, arguing that piracy is an important trope in the tale in that it recalls seventeenth-century piracy between the Caribbean and the Pacific, on the one hand, and anticipates the shift of actual piracy and slavery to the Pacific realm toward the end of the century, on the other. In what follows, I highlight points of connection and translation between Melville’s Atlantic and Pacific imagination rather than presenting a purely (trans)Pacific reading of “Benito Cereno,” which would have to situate itself more precisely within the fledgling field of (trans)Pacific studies.^3^ I am drawing on a transoceanic framework of enquiry suggested by Michelle Burnham (among others), which defies earlier Area studies conceptions of the Atlantic and the Pacific that rest on essentialist assumptions regarding a region’s history, culture, and identity. This is not to imply, however, that the Pacific and the Atlantic are unmarked by different geographical, material, economic, and socio-political conditions and developments, but to investigate how trans-Atlantic epistemologies (e.g. with regard to race and legitimacy) entered the Pacific in the US imperialist context.

“Benito Cereno” is prophetic regarding the violent imperial scenarios that would come to characterize US–Pacific relations in this period, with America “Pushing Out Pacific Frontiers”^4^ already since mid-century and creating what John Eperjesi calls “the American Pacific,” an economic and cultural frontier, both politically and in literature. Eperjesi defines the American Pacific as “the unstable ideological terrain upon which struggles over the scope and direction of economic and territorial expansion have been historically waged,” “a regional, or regionalizing [Orientalist] myth,” and “a space of conflict,”^5^ with the possessive *American* expressing the “will to mastery and control that has led to the Americanization of the Pacific,”^6^ and continuing a providential national design eventually leading to the “Pacific Century.”^7^ Arif Dirlik’s concept of the “Euro-American Pacific” (1992) likewise emphasizes transnational exchanges, alliances, and rivalries fundamental for the spread of capitalist relations in the Pacific realm. Just as the pursuit of the sperm whale “began in the Atlantic and gradually pulled American whalers up the coast of Chile and then across the Pacific”^8^ and as the gruesome Pacific sealing industry took off,^9^ the African (and later Pacific Islander) slave trade entered this maritime region.

In “Benito Cereno,” the colonial Atlantic becomes metaphorical to the degree that conflictuous power relations among Old and New World agents as well as their (enslaved) Others are carried from one ocean to another. At the same time, the novella remains historically grounded as it re-constructs these Atlantic relations in the Pacific in a historically specific hemispheric continuum, a “transoceanic imaginary” of slavery and piracy.^10^ Unlike the myth-building texts that Eperjesi examines, which “instance a will to imagine Asia and the Pacific as constituting a unified and structured region,”^11^ “Benito Cereno” imagines the Pacific as more complicated, a space of contradiction beyond stable colonial binaries, between metaphor and historicity, between possibility (for revolt and the renegotiation of justice/legitimacy; for a black Pacific counterculture) and the continuity of unequal, racialized power relations – resembling Melville’s manifold discussions of slavery as both a universal, Hegelian master-slave relation and as historically and geographically localizable.^12^

## The black Atlantic/Pacific

Propelling a turn to oceans (and more recently, archipelagoes)^13^ in American studies, Gilroy’s black Atlantic concept has been used to re-write American literary/cultural history, advancing the maritime imagination as a fundamental, yet neglected aspect of American culture. In *The Black Atlantic: Modernity and Double Consciousness* (1993), Gilroy reads the sea in a double-edged manner as a fluid space of both exploitation and resistance. “[T]he image of ships in motion across the spaces between Europe, America, Africa, and the Caribbean”^14^ is evoked to highlight transnational movement within the black diaspora, recalling the Middle Passage, but also subsequent travels of Afrodiasporic individuals and activists such as Martin Delany or Frederick Douglass. Further, Gilroy foregrounds “the circulation of ideas […] as well as the movement of key cultural and political artefacts” as highly important means of cultural and political exchange within the black Atlantic world.^15^

Though Gilroy’s concept has been highly criticized, it has also been a productive framework for Atlantic/American studies.^16^ On the side of the critics, Gilroy has been denigrated for overlooking two entire continents, Africa and Latin America,^17^ as well as the Pacific and Indian diasporas, in his grandiose claims about an Afrodiasporic Atlantic counterculture;^18^ the concept has been further attacked for its lack of gendered critique^19^ and its all-too-celebratory esthetics of transnational movement and fluidity that may be complicit with the capitalist “power structures it aims to resist.”^20^

With regard to its usefulness as a concept for this article, the black Atlantic has recently been adapted for (trans)Pacific American studies. Laudably, these adaptations constitute a branching-out of the concept in many ways rather than a uniform translation of Gilroy’s idea onto another ocean. The term “black Pacific” was first used by Eric Gardner in a study of the black (US-)American West to describe a migratory pattern of African Americans moving to California, either by ship around Cape Horn or by land across the Isthmus of Panama. Gardner relates the term to the black Atlantic, viewing Gilroy’s concept positively as highlighting a “set of power relations between a collection of regions in dialogue.”^21^ Currently, conceptualizations of a black Pacific in order to “initiate an oceanic dialogue bridging the black Atlantic and the Indo-Pacific” can be found in Francophone studies (for example, in a special issue of the *International Journal of Francophone Studies* from which this quote is taken),^22^ in ethnological studies (for example, Shilliam, who uses the term to refer to “the variable, shifting nature of African-American and North-Pacific Asian ties of kinship, politics and ideology against the backdrop of US imperial ambitions”),^23^ and in various contexts of African American studies.^24^ Smyth summarizes further usages of the black Pacific “to describe black cultures on the Pacific side of South America […], African American cultures in Japan, Hawai’i, and the Philippines […], and Afro-Amerasians in America.”^25^ It exceeds the scope of this essay (and would certainly warrant an article of its own) to compare the various conceptions and usages of the black Pacific concept, but what they have in common “is a sense of different cultural diasporas crossing each other, and a sense of unexpectedness, of being ‘off-route’ and in new territory” – in what Smyth aptly terms “awkward adjacencies.”^26^

Unlike the black Atlantic, the black Pacific model has not (yet) made it into Melville studies. Following Heidi Feldman, my analysis of “Benito Cereno” uses the term as referring to “a newly imagined diasporic community on the periphery of the black Atlantic” that, in this case, harks back to Atlantic-Pacific genealogies of piracy and slavery.^27^ As I will show, the black Pacific inhabits a similarly ambivalent space in terms of identification as Gilroy’s black Atlantic in Melville’s novella: Feldman claims that unlike black Atlantic double consciousness, which – at least in Gilroy’s version – is a result of “dual identification with pre-modern Africa and the modern West,” the black Pacific “negotiates ambiguous relationships with local creole and indigenous cultures and with the black Atlantic itself,” emphasizing a relationality of crucial importance in “Benito Cereno.”^28^ My use of the term is also indebted to Feldman’s pointed critique of Gilroy, who argues that the black Atlantic fails “to encompass adequately the experience of the black Pacific, where enslaved Africans were forced to continue parts of their voyage […], leaving the Atlantic Ocean behind.”^29^

Nevertheless, using the term “black Pacific” also means to acknowledge a continuum with the black Atlantic paradigm with regard to the countercultural, diasporic effects of the Middle Passage that “Benito Cereno” stages in the form of a slave revolt and mutiny, on the one hand; on the other, the black Pacific model, while far from thoroughly theorized, serves as a heuristic tool in order to tease out the interrelations between the Atlantic and Pacific that the novella establishes. In the following, I offer a reading of the tale with a focus on the two main literary strategies through which the Atlantic slave trade correlates with a fledgling US imperialism in the Pacific: its spatiality and geographic imagination and its building on historical references to exploration, mutiny, and piracy.

## “Benito Cereno” and the shadows of piracy

In 1928, Harold Scudder discovered that Amasa Delano, a distant ancestor of Franklin Delano Roosevelt and historical captain and explorer, and his *Narrative of Voyages and Travels in the Northern and Southern Hemispheres: Comprising Three Voyages round the World; Together with a Voyage of Survey and Discovery, in the Pacific Ocean and Oriental Islands* (1817, edited by Eleanor Roosevelt Seagraves in 1994) formed the basis for Melville’s tale. In chapter 18, Delano recounts how his ship *Perseverance* encountered the Spanish *Tryal* near the small, uninhabited desert island of Santa María off the coast of Chile in 1805. Its slaves had been transported from Senegambia to Buenos Aires, marched across the pampa to Lima, “the radiating heart of the black Pacific,” where they embarked on the *Tryal* and subsequently overthrew the Spanish crew.^30^ Melville re-wrote the chapter and also included a portion of one of the legal documents appended, omitting some passages but also making additions; notably, Melville’s father-in-law, Lemuel Shaw, acted as Delano’s lawyer when the captain was later sued by his creditors.

The opening description of the setting is highly significant: Melville displaces the island of Santa María, moving it from off the coast of central Chile “toward the southern extremity of the long coast of Chili.”^31^ Will Slocombe interprets this move as perhaps “a sign for the continued southwards expansion into Mapuche territories that was ongoing at the time,”^32^ but more likely as signifying a euphemism for “southern [racist] extremism.” Following Slocombe, the tale turns the Pacific into an important site for the slavery debate right away:

*Rather than losing or misplacing the island, the politics of Spanish-governed Chile becomes Melville’s catalyst for a transposition of the race debate, elided in Delano’s original Voyages into American consciousness*.^33^

Melville changes the year of the revolt from 1805, the heyday of slavery in Uruguay, Argentina, and other South American countries,^34^ to the “crisis year of 1799,”^35^ characterized by Toussaint L’Ouverture’s triumph in the revolution of Santo Domingo (Haiti) and concomitant, ever-increasing fears of slave uprisings and “black empires” throughout the Americas.^36^ In this, he displaces enslavement for rebellion and projects a Pacific story back into an Atlantic-Caribbean historical frame. The year of the novella’s publication in the anti-slavery journal *Putnam’s Monthly* is equally significant, coinciding with the Fugitive Slave Act (1850) and its silencing effects on sympathizers of abolition, and the Kansas-Nebraska Act (1854) that opened the west of the United States for the expansion of slavery toward the Pacific.^37^ “Benito Cereno” comments on this period of crisis^38^ as well as on the *Amistad* case of 1839 in the Caribbean Sea, in the context of which defense lawyer John Quincy Adams justified slave revolt at sea in terms of the natural right to liberty and revolution, explicitly dissociating the mutineers from accusations of piracy.^39^ Territorial, positive law, abolitionists such as Adams argued, did not translate to non-territorial waters. In the case of the *Amistad*, Justice Joseph Story eventually asserted that as much as the slaves might have committed dreadful acts, “they cannot be deemed pirates and robbers in the sense of the law of nations.”^40^ For him, it was the slave ship that was to be characterized as piratical ever since the slave trade had become illegal in international law. The *Amistad* case strengthened and inspired discourses that saw the topics of piracy and slavery in need of disentanglement or reversal – the slavers rather than mutinous slaves needed to be unmasked, from the abolitionist perspective, as the “real” pirates. These widely reported legal arguments are subtly negotiated through literature in “Benito Cereno.”

The *San Dominick* and its crew confront three historical positions, associated with three continents:^41^ Old World (represented by Columbus’ figurehead, references to Benedict de Las Casas, who introduced African slavery in the New World, and to the Inquisition, echoed in the name Benito and the evocation of “Black Friars” [36]);^42^ African (Babo and his fellow enslaved mutineers in a state of historical transition, entering the American hemisphere); and New World (conflating it with Anglo-America by its representative Delano). In this, “Melville’s tale […] anticipates […] an explosive resolution of the conflict between American democracy, Old World despotism, and [black] New World revolution.”^43^ An allegory for the theatrical stage as well as for “allegory with a black Atlantic counterculture that, paradoxically, is formed in the Pacific,”^44^ the *San Dominick* displaces this national(ist) allegory with a black Atlantic counterculture that, paradoxically, is formed in the Pacific.^45^

Lima, the closing geographical setting of the novella, is a “standard trope for [Old World, Spanish-colonial] corruption” in Melville’s work, as Amy Kaplan remarks.^46^ In his biography of Melville, Joshua Parker even claims that “no other city in the Americas ever caught and haunted his imagination as Lima did” after he visited it in 1844,^47^ as a “site from which to engage romantic [Anglo-]American historiography,” highlighting its “emerging significance in American cultural dialogue.”^48^ Most notable among Melville’s many references to Lima as a city of decay and desolation, “The Town-Ho’s Story” (chapter 54 of *Moby-Dick*), has Dons from Lima ask about Lake Erie. When they are presented with the image of a waterway of global significance, they exclaim: “No need to travel! The world’s one Lima.”^49^ With Lima representing corruption and the world represented synecdochally by Lima, its mention in “Benito Cereno” ten times – along with Cape Horn (eleven times), the most referenced place – connects the past violence of Spanish colonization and the introduction of slavery to the Americas with its future in the Pacific. The novella’s ending in the Lima Plaza remains with the reader as a space of racial violence as Babo is tried and decapitated there, his head “fixed on a pole in the Plaza” where it “met, unabashed, the gaze of the whites” (102), his “sightless gaze […] forcefully challeng[ing] their supremacy” beyond the narrated time.^50^ The story closes by emphasizing

[…] the evil of colonial power, of court-sanctioned slavery, of mutilating violence against the oppressed […]. In the plaza, one sees them only too clearly [as] the stage from which Babo can make them visible.^51^

Oddly, the tale thus both highlights Peru as a crucial Pacific agent in the context of the South American trade to Manila during the formation of the early modern Pacific,^52^ including transpacific slave traffic,^53^ and foreshadows Peruvian slavery in Polynesia in the early 1860s^54^ as well as, more generally, the rise of slavery in the South Seas after the Civil War, when there was an increased labor demand to produce cotton, rice, and other goods.^55^ US slave traders were “instrumental in the propelling of the horrendous Peruvian Slave Trade in Polynesia,”^56^ historian Gerald Horne notes; ever moving westward, a “White Pacific/White Atlantic of planters” did not stop at the Pacific coast – paradoxically at the same time as African Americans looked to the Pacific as a sanctuary, giving rise to a black Pacific counterculture.^57^ Blackbirding, the raiding of Melanesian and Polynesian coasts for slaves, represented a “trans-Pacific fate” with the rise of white supremacy in the Pacific since mid-century.^58^ Indigenous populations were racialized as colored already at the end of the eighteenth century, which facilitated rationalizing their enslavement, a fact that the historical Amasa Delano discusses in his *Narrative* in an episode on New Guinea, whose inhabitants he describes as “Negroes” that are

[…] well known to hate white people so much as to reward an individual, by making him a chief when he will bring them a white man’s head [because] Europeans seized and carried them away as slaves, in a most treacherous manner.^59^

Initially, the imagery in “Benito Cereno” transforms the black Atlantic into a *gray* Pacific, in which perception is always already tainted, blurred by Delano’s underlying racist and exceptionalist cultural framework. Accordingly, the color imagery centers on the color gray, mentioned four times in the description of the atmosphere in the opening paragraph:

Everything was mute and calm; everything gray […]. The sky seemed a gray surtout. Flights of troubled gray fowl, kith and kin with flights of troubled gray vapors among which they were mixed, skimmed low and fitfully over the waters […]. Shadows present, foreshadowing deeper shadows to come. (35)^60^

The events aboard the *Tryal* are rendered in terms of ambivalence as grayness “envelops the appearance of the mysterious San Dominick.”^61^ The muddying color gray and shadow imagery – “the Negro […] an absence that casts a shadowy presence”^62^ – marks the tale, from its inception, as revolving around clarity of perception and its difficulties in a Pacific setting marked by slavery; while the colors white and black, in a reversal of the dominant racial binary, are mainly associated with death and health, respectively.^63^

Sterling Stuckey and Joshua Leslie were perhaps the first to focus on Melville’s highly significant forms of evoking piracy in “Benito Cereno,” complementing the novella and its historical source with archival documents from the Archivo Nacional of Santiago de Chile.^64^ They demonstrate that, unlike Melville’s story, Delano’s narrative explicitly includes the accusations of piracy against him in court, presenting them as a vile stratagem by the ungrateful Spaniards. For Stuckey and Leslie, Delano “embodied the spirit of imperialism” that, in search of wealth, reduces “moral scruples” to a mere “petty annoyance”^65^: they cite the deposition of Delano’s crew member Peter Samson, according to whom the crew “did not know whether, having wasted the voyage, the captain would practice piracy in order to meet the expenses of the expedition.” Melville’s reversal of the charge, placing it upon the Spaniard, seems “harsh,” “almost shocking,” “Delano’s suspicions regarding Benito Cereno bordering on paranoia.”^66^ In the original documents, it is Benito Cereno who calls Delano a piratical “monster”; Melville obliterates the charge and instead has Delano call Benito a murderer.^67^ Thus,

[b]asing the theme of piracy in the novella on the Peter Samson charge to which Delano refers but does not include […], Melville extends the conflict between the historical Delano and Benito Cereno into the very heart of the novella.^68^

Delano himself suppressed some of the documents available in the Archivo in his record of the revolt and its aftermath; at first sight, “Benito Cereno” certainly concurs with that suppression, omitting accusations of piracy against Delano. Yet it is important here to distinguish between Delano’s and the auctorial narrative perspective: in the parts that are told from Delano’s unreliable and limited point of view, it is no surprise that he is disassociated from any such suspicion. But even in these parts a shadow of piracy is subtly cast on Delano. For one, he uses the same motivational rhetoric as the buccaneers and Caribbean pirates of the seventeenth century, a rhetoric of plunder that does not distinguish between gold, silver, and human “cargo”:

The more to encourage the sailors, they were told that the Spanish captain considered his ship good as lost; that she and her cargo, including some gold and silver, were worth more than a thousand doubloons. Take her, and no small part should be theirs. (87)

As Gesa Mackenthun states, Delano’s attack on the *San Dominick* “reveals him as a figure that spans the period from the history of seventeenth-century buccaneers to the more subtle piracies of the 1850s,”^69^ representing him “less as an honorable merchantman than a privateer and pirate”^70^ whose chief mate, it is stated explicitly, also had been a “privateer’s-man, and, as his enemies whispered, a pirate” (87; a fact not mentioned in Delano’s *Narrative*).^71^ This presents one instance in which the text has the fictional Delano externalize the accusation of piracy faced by the historical Delano, who indeed worked as a privateer during the American Revolution.

Melville’s tale further associates Delano with piracy by renaming the historical Amasa Delano’s ship *Perseverance* the *Bachelor’s Delight*,^72^ referencing a famous pirate ship which had originally been a Danish slaver and was renamed by William Dampier (it is also the name of one of the two ships hailed by the *Pequod* in chapter 115 of *Moby-Dick*). Notably, Dampier, along with Lionel Wafer, Basil Ringrose, James Kelley (a later associate of Captain William Kidd), and dozens of others, opened up the Pacific via the coast of Panama in the late seventeenth century for exploration, piracy, and Anglo trade. In 1683, Dampier and his crew had gone to Guinea, became involved in the slave trade, and took a number of enslaved African women whom they abused in every possible way on the ship.^73^ Dampier’s own *A New Voyage Round the World* (1697) also presents an account of the first contact with coastal and island indigenous populations of the Pacific, hence constituting an early moment of inscribing the region in terms of the European-Atlantic “incursion upon indigenous culture” on the other side of the continent.^74^ In “The Encantadas; or, Enchanted Isles,” another of Melville’s 1856 *Piazza Tales*, first published two years earlier in *Putnam’s*, Melville discusses Dampier on the Galápagos as an exemplary pirate between utopian and dystopian representation in “Sketch Sixth: Barrington Isle and the Buccaneers”:

That the buccaneers perpetrated the greatest outrages is very true; that some of them were mere cut-throats is not to be denied: but we know that here and there among their host was a Dampier, a Wafer, and a Cowley, and likewise other men, whose worst reproach was their desperate fortunes – whom persecution, or adversity, or secret and unavengeable wrongs, had driven from Christian society to seek the melancholy solitude or the guilty adventures of the sea […]. [A]ll of the buccaneers were not unmitigated monsters. […] Could it be possible, that they robbed and murdered one day, reveled the next, and rested themselves by turning meditative philosophers, rural poets and seat builders the third? […] Still, strange as it may seem, I must also abide by the more charitable thought, namely, that among these adventurers were some gentlemanly, companionable souls, capable of genuine tranquility and virtue.^75^

Again, Melville refuses “a unified political or ideological position” here, as marauders are transformed into pastoral poets in a Deleuzian “archipelago-perspectivism,”^76^ including geographical and natural historical accounts combined into a fragmentary and incomplete narrative.^77^ Atlantic piracy, in its double nature as outlaw resistance and colonial complicity, translates onto the Galápagos in the Pacific as an area off the map, a safe haven and an archipelago of escape: the pirates swerve between being masterless men and becoming masters themselves as vanguard colonizers, like the whalers, sealers, and fur traders in the North Pacific, paving the way for Pacific trade and subsequent US-imperialism.^78^ They represent the vexed mirror image of a fledgling, transoceanic capitalist modernity by envisioning a Pacific-archipelagic paradise built on the exploitation of African and indigenous Pacific populations. Ravaging the “opulent countries” of “the Pacific side of the Spanish colonies,” they are rewarded with “silks of Asia” and a life of tranquility on the Galápagos in “The Encantadas.”^79^ Spatiality in “Benito Cereno,” translating a Middle Passage scenario of enslavement and a slave revolt into a Pacific world of bleak prospects, contrasts with this opening of democratic potentialities in the Galápagos in “The Encantadas.” The latter’s pirate-based “permanent *Riotocracy*, which gloried in having no law but lawlessness”^80^ at the same time welcomes “any fugitive” and “each runaway tar” and in this way imagines what Amy Kaplan calls “future modes of confederating”^81^ – without being eventually hanged; in comparison, “Benito Cereno” is much less optimistic about the utopian possibilities of the Pacific realm.

In addition, Delano’s boat is called *The Rover*, and though he references the vessel frequently when he seeks out a stable sign, reassured by its familiarity – he metaphorically compares her, like “the Negro” a little later, to a Newfoundland dog (64) – its linguistic signification carries instability and fluidity. Besides referencing a loyal pet, the dog metaphor at the same time evokes the piratical English “sea-dogs” of the sixteenth century (most notably Francis Drake), and a rover demarcates mobility and inconstancy, the shiftiness of the sea and of ships themselves roving between various owners – slave traders, merchantmen, pirates.^82^

By associating the enterprising US-American captain with piracy, then, “Benito Cereno” also implies that Manifest Destiny, epitomized on America’s maritime frontiers by the historical Delano and his filibustering peers (who attempted to expand the US political sphere towards Cuba, Haiti, Central America, and the Pacific), is merely “the rhetorical camouflage for a largely ‘piratical’ enterprise,”^83^ invalidating the distinction between American expansionism and European colonialism.^84^ The characterization of Spaniards and Africans as potential pirates serves as another camouflaging strategy, a reversal by use of the ideological category of piracy to stereotype others, which externalizes illegitimacy and illegality, projecting them from the USA onto such others:

Just as Delano projects onto Cereno piratical schemes that he himself will blithely – and with the best intentions – actually perpetrate, so the United States in the mid-nineteenth century was projecting onto the decaying Spanish empire images that would legitimize its own piratical – but of course well-intentioned – expansionism.^85^

In sum, Melville does not obliterate piracy as a potential status of the American captain but rather recasts it as a “kaleidoscopic question about who was the real pirate.”^86^ When Delano is suspicious about, but refrains from interpreting, the amount of various national flags he discovers in a locker (“exposing various colored bunting, some rolled up, others half unrolled, still others tumbled,” 70), the reader familiar with earlier Caribbean pirate narratives, which reported that buccaneers used any national flag if it was to their advantage, cannot but read this discovery as a re-writing of Benito Cereno’s trading in slaves as “piracy.” The flags, cast together in disorderly manner, also function as a transnational metaphor for the messiness of the slave trade, acted out under all of these flags and hence associating all of these nations with piracy. In the words of Eric Sundquist, “Benito Cereno” highlights that “slavery was hemispheric and that its fullest literary representation as well as its fullest political critique required a view that embraced several cultures, several nations.”^87^ In this context, Babo’s misuse of the Spanish flag as a shaving towel in the tale’s most famous scene can be read as another reversal, suggesting that this misuse is much less criminal or “piratical,” for that matter, than the slave trade committed under all of these “bright colors” (71). Babo and his peers are similar to pirates in that the black slave is by law positioned outside the community of humankind, of “civilized,” lawful nations as the silent, barbarous, savage other: “nothing done to them can be a violation of law” and “they therefore have no ‘natural’ right to self-defense.”^88^

Delano’s earlier suspicion that the *San Dominick* “be of a piratical character” (56) displays the way his perception is shaped by a benevolent racism, as he sees Spaniards as shady characters but Africans as “too stupid” (63) to be the masterminds of a piratical scheme, taking to them “not philanthropically, but genially, just as other men to Newfoundland dogs” (63) and thereby creating a “fiction of fidelity.”^89^ Blinded by the imperial conflict between the Spanish and Anglo Empires in the New World and American exceptionalism,^90^ Delano at first suspects Cereno rather than the Africans as potentially piratical, crying out in a double alliteration that “this plotting pirate means murder!” (84). Likewise, he is blinded by his self-righteousness and alleged innocence as a “Jack of the Beach” (64), musing “I to be murdered here at the ends of the earth, on board a haunted pirate-ship by a horrible Spaniard? Too nonsensical to think of! Who would murder Amasa Delano?” (64); the “pirate ship” is a characterization that is in fact much more accurate with regard to Delano’s vessel.^91^ It is the black mutineers’ use of a fine-tuned arrangement of deceptive behavior, their only available weapon, that subsequently associates them with the deceptive techniques of Caribbean and other historical pirates (such as their mis/use of national flags).

## A bleak Pacific

In one of a number of suspicious moments, the narrator, through the eyes of Delano, compares the black mutineers specifically to “Malay pirates” for whom

it was no unusual thing to lure ships after them into their treacherous harbors, or entice boarders from a declared enemy at sea, by the spectacle of thinly manned or vacant decks, beneath which prowled a hundred spears with yellow arms ready to upthrust them through the mats. (56)

That the comparison relates to Pacific rather than Atlantic piracy, Malay rather than Caribbean pirates, references the historical Delano’s New Guinea chapter, in which he recounts an attempt to enter an area of New Guinea occupied by Malays, whose past experiences with white men (and their history of abusing Malay women), has made them hostile.^92^ In “The Grand Armada” (chapter 87 of *Moby-Dick)*, Melville already portrayed “the piratical proas [canoe-like boats] of the Malays” as possessing political agency and a sense of geo-strategic power, also with regard to indigenous people resorting to piracy in order to defend themselves from colonizing agents.^93^ While the historical Delano gets into an argument with the Malays, the fictional Delano immediately suppresses his suspicions: “Not that Captain Delano had entirely credited such things. He had heard of them – and now, as stories, they recurred” (56). Only upon being openly attacked at the climax of the action does the discourse of piracy return, as he perceives the slaves, “now with the scales dropped from his eyes […] with masks torn away, flourishing hatchets, and knives, in ferocious piratical revolt” (85); his surprise at this turn of events indicates that he was “duped, perhaps more by his own perceptions than by the rebels’ acting.”^94^

Haegert notes that in the process of repeatedly suppressing suspicion about the power relations aboard the *San Dominick*, Delano “unfailingly asserts a cultural and racial hegemony which masks itself as a kind of natural law” as he time and again reverts to a blind belief in “the ever-watchful Providence” (83),^95^ assumed to be on the side of its US-American agents. His Puritan sense of mission, articulated also by presenting him as a figure whose “conscience is clean” (64), is exposed by Melville as an unjustified fiction, as he highlights Yankee complicity with Old World orders (embodied by Cereno) and the institution of slavery, drawing a bleak picture of the Pacific.^96^ In its construction of “transracial savagery,”^97^ then, “Benito Cereno” nihilistically suggests that both whites and blacks are potential pirates, but Delano’s association with piracy also implies that American leadership (especially regarding Atlantic/Pacific trade) – the tale’s de-facto result – often originates in illegitimate acts.

Highlighting its Pacific dimension is not to imply that Melville’s narrative is not still, thematically, Atlantic, with the transatlantic slave trade “quietly govern[ing] the story.”^98^ “Benito Cereno” is indeed both an Atlantic and a Pacific text – the latter, after all, Melville’s preferred scenario for his maritime tales – demonstrating that New World slavery and its specters transgress geographical borders. Following Hester Blum, the precariousness of national and individual sovereignty in the Atlantic world always already haunts the Pacific like a specter.^99^ “Benito Cereno,” directed at a cisatlantic market,^100^ presents an “explicitly transnational fictional perspective […] for the negotiation of conflict lines, historical controversies and predicaments of US society and culture,”^101^ its “hemispheric dimensions mak[ing] the novella a Pan-American literary endeavor.”^102^ Similarly, Susan Gillman and Kirsten Silva Gruesz have read the novella as Melville’s “most overtly hemispheric work” as it maps out “a New World or ‘Americas’ history of race-slavery and colonialism, revolt and revolution.”^103^

Melville’s novella, a Gothic tale of haunting and enchantment pervaded by the imagery of death, ghosts, and a deep sense of gloom (e.g. 38, 55, 62), creates a bleak rather than black Atlantic/Pacific continuum.^104^ Reaching the Pacific, the black Atlantic, as a counterculture of resistance against Western notions of culture, has turned into a bleak version of itself. Scholars have criticized that the novella leaves no space for utopian possibility or “remedial action,”^105^ “no […] hope about the fruitful merging of cultures”;^106^ “[b]ecause of the question of race, we are *not* saved, we remain in the shadow”^107^ in an “aprogressive narrative[] of modernity”^108^ that resists closure and historical teleology.^109^ Emancipation and free selves are exposed as always already tainted by a global history of institutionalized slavery and piracy that even the American captain cannot escape.^110^ Any notion of a New World utopia is rendered null and void, as “past, present, and future seemed one” (85),^111^ in a

convulsive history of the entire region and epoch – from the Columbian discoveries of the Americas, through the democratic revolutions in the United States, Haiti, and Latin America, to the contemporary crisis over the expansion of the “Slave Power” in the United States.^112^

The tale yokes together Europe, Africa, the Atlantic, the Americas, and the Pacific as tainted by the institution of slavery and by piracy.^113^ As Lawrie Balfour concludes, the novella’s “great, troubling success may consist most dramatically in its capacity to foreshadow dangers that would linger long *after* the storm.”^114^ In this light, US-American perceptions of itself as exceptional in, and pushing forward, world history appear as self-complacent, continuing to repress the shadow of “the negro” (101) while simultaneously emulating the hegemonic ambitions of Old World powers.^115^ Indeed, “Benito Cereno” asks a fundamental question about slavery that cannot be limited to any national or racial context and hence is both a central question for the Atlantic and the Pacific: “what in human relations gives rise to slavery and what slavery gives rise to in human relations.”^116^

## Conclusion: “Benito Cereno” – Triangulating the Pacific

Charles Olson famously called Herman Melville a “Pacific Man,”^117^ and yet Melville studies have rarely connected Melville’s popular early Pacific writings (such as *Typee* and *Omoo*) and his later, stylistically and philosophically more elaborate works. In my view, “Benito Cereno” does that work of conjunction. It revises Melville’s earlier Pacific descriptions of racial difference,^118^ allegorizing the Pacific as primarily a space in which Western civilization (mis-)perceives itself.^119^ As Melville was unable to return to a safe “white Atlantic” (Horne)^120^ after his Pacific travels, he came, forever after, to reflect on the epistemological consequences of cultural contact, difference, and imperialism in light of the Americanization of the Pacific. “Benito Cereno” is located between the gray Atlantic and the bleak Pacific, in what Yunte Huang has called, quoting Melville (who himself borrowed the term from the Scottish poet Thomas Campbell’s “The Battle of the Baltic” of 1809), “the deadly space between”:^121^ which indicates the

deadly inadequacy of human language to measure the gap; […] his resort to metaphors exemplifies not only the deadliness of the geopolitical reality that constantly escapes the grasp of language, but also the slipperiness and ambiguity of the space between […] history and literature.^122^

Like *Moby-Dick*, “Benito Cereno” “links and crosses the Pacific as an oceanic American plantation,” enacting a “troubled crossing of the Atlantic into New World real estate.”^123^ The novella’s Pacific triangle, continuing the socio-cultural and political effectuality of the triangular trade, reaches back to the Panamanian isthmus, which was crossed in the late seventeenth century by Anglo pirates like Dampier, to Cape Horn as the main maritime entry point to the Pacific, and to Lima as one of the centers of Pacific slavery. While the novella negotiates national tensions in the 1850s, “recast[ing] the collective consciousness of the nation through a critique of New World slavery,”^124^ its geography can “help us to re-encounter the histories of those Pacific shores in ways that make the kinds of problems represented […] by the ‘Atlantic’ more than a specter.”^125^ In this, Melville creates a transoceanic, Atlantic-Pacific imaginary deadlocked between the roots and routes of slavery.

## Notes

1 For example, Sundquist, Mackenthun.

2 One notable exception is Gniadek’s autobiographically inspired essay “Americans Abroad: Melville and Pacific Perspectives,” which critically reflects the imperialist dangers of the transatlantic and transnational turn in American studies.

3 I am referring to recent work by historians Matt Matsuda and David Igler in the context of Pacific studies and by Huang, Hoskins and Nguyen, Cruz, Duerr and Schorch, and Suzuki for transpacific studies.

4 Perry, *Facing West*, 59; Eperjesi, *Imperialist Imaginary*, 22.

5 Eperjesi, *Imperialist Imaginary*, 4.

6 Ibid., 14.

7 Huang, *Transpacific Imaginations*, 6, 73.

8 Eperjesi, *Imperialist Imaginary*, 47.

9 Grandin, *Empire of Necessity*, ch. 13, 14.

10 DeLoughrey, *Roots and Routes*, 4. She uses the term in the context of island literatures.

11 Eperjesi, *Imperialist Imaginary*, 16.

12 Grandin summarizes this relation between existential slavery and slavery as a Western institution: “[I]t is exactly Melville's existential digressions that speak directly to the problem of slavery in Western society, that go straight to the heart of what the massive and systemic subordination of millions and millions of human beings over the course of hundreds and hundreds of years meant to the societies that prospered from slavery and to the slaves who suffered creating that prosperity” (*Empire of Necessity*, 125; see also 54, 89).

13 See Roberts and Stephens, “Archipelagic American Studies”; DeLoughrey, *Roots and Routes*.

14 Gilroy, *Black Atlantic*, 4.

15 Ibid., 4.

16 For an overview, see Evans, “Black Atlantic.”

17 Goebel and Schabio, “Introduction”; Feldman, “Black Pacific,” 206.

18 Evans, “Black Atlantic,” 255.

19 Reid-Pharr, “Engendering the Black Atlantic.”

20 Evans, “Black Atlantic,” 266; Dayan, “Middle Passage as Metaphor.”

21 Neary, “Mining the African American Literary Tradition,” 367, n. 22.

22 De Souza and Murdoch, “Oceanic Dialogues,” 223.

23 Shilliam, *Black Pacific*, 9.

24 See Neary’s study on black miners in California, Smyth on Caribbean poetics, Feldman’s musicological essay on the Afro-Peruvian revival, Hudson on black artists from Vancouver, Taketani on James Weldon Johnson.

25 Smyth, “Black Atlantic,” 390.

26 Ibid., 391. Likewise, George Lipsitz points out the contradictions that he sees inherent in a black Pacific model as similarly awkward when looking at African American-Japanese alliances that embraced Japanese fascism and imperialism (“‘Frantic to Join’,” 350).

27 Feldman, “Black Pacific,” 206.

28 Ibid.

29 Ibid., 207.

30 Grandin, *Empire of Necessity*, 239. This is arguably the most detailed and well-balanced historical study of Delano and the slaves aboard the *Tryal*.

31 Melville, “Benito Cereno,” 34. Subsequent references to the novel are cited parenthetically.

32 Slocombe, “Santa Maria,” 31.

33 Ibid., 32.

34 Grandin, *Empire of Necessity*, 7.

35 Levine, *Conspiracy and Romance*, 213.

36 Sundquist, “New World Slavery,” 95–96; Levine, *Conspiracy and Romance*, 168–170; Fortin “‘Excited Almost to Madness’.” Mackenthun reads Babo as “a composite figure of the historical characters Singue, leader of the *Amistad* revolt, and Toussaint L’Ouverture […] mixed with traces of African trickster figures,” *Fictions*, 125.

37 Goldberg, “Mute Testimony,” 7.

38 Sundquist, “New World Slavery,” 94.

39 Rogin, *Subversive Genealogy*, 211. Carolyn Karcher explains how the trope of piracy connects “Benito Cereno” and the *Amistad* case: “Charges of piracy were central to the trial. The press had repeatedly described the *Amistad* as a pirate ship when it was sighted […], but the Africans’ defense team […] successfully argued that it was not piracy for persons to rise up against those who illegally held them captive” (“Notes,” 2465). See also Thomas, *Cross-Examinations*, 108.

40 Qtd. in Rogin, *Subversive Genealogy*, 212.

41 Karcher, *Shadow Over the Promised Land*, 136.

42 On the parallel construction of the American South and the Catholic Spanish Empire in “Benito Cereno,” see Levine, *Conspiracy and Romance*, 202.

43 Sundquist, “New World Slavery,” 106.

44 Levine, *Conspiracy and Romance*, 191.

45 Reichardt, *Alterität und Geschichte*, 128, following Gilroy.

46 Kaplan, “Transnational Melville,” 45.

47 Parker, *Herman Melville*, 281. In the 1840s, Melville visited many of the same places Delano had been to 40 years earlier; Grandin, *Empire of Necessity*, 166.

48 Kelley, “Style of Lima,” 62–63.

49 Melville, *Moby-Dick*, 1061.

50 Ibid., 68.

51 Ibid., 69.

52 Wilson, *Reimagining the American Pacific*, 58.

53 See Tardieu, “Origins of the Slaves.”

54 Maude, *Slavers in Paradise*; Huang, *Transpacific Imaginations*, 68.

55 Horne, *White Pacific*, 2, 37.

56 Ibid., 5.

57 Ibid., 10 and ch. 8; on the British slave trade in the Pacific between 1864 and 1908, see Phillips, “Mapping Imagination.”

58 Horne, *White Pacific*, 11 and ch. 1; Huang, *Transpacific Imaginations*, 68.

59 Delano, *Narrative of Voyages and Travels*, 48.

60 This description is one of Melville's deliberate alterations: Grandin notes that Delano’s historical encounter with the *Tryal* took place on a sunny day; *Empire*, 213.

61 Mackenthun, *Fictions of the Black Atlantic*, 117; Nnolim, *Study in Meaning of Name Symbolism*, 57.

62 Levine, *Conspiracy and Romance*, 222.

63 Richardson, *Interpretation With Annotated Text*, 211 n. 31.

64 Franklin reveals that the historical Delano was, in fact, a privateer and likewise emphasizes that he was regarded as a pirate by his enemies; “Past, Present, and Future Seemed,” 237.

65 Stuckey and Leslie, “Aftermath.”

66 Ibid., 271; also Grandin, *Empire of Necessity*, 231.

67 Stuckey and Leslie, “Aftermath,” 271.

68 Ibid., 273.

69 Mackenthun, *Fictions of the Black Atlantic*, 123.

70 Ibid., 115; Nnolim, *Study in Meaning of Name Symbolism*, 17.

71 Emery, “Manifest Destiny,” 102. Melville adds the explicit reference to piracy to the first edition of *The Piazza Tales*. In the original serial publication in *Putnam's*, he is just “a privateer's-man – to head the party” (see Richardson's concordance, *Interpretation With Annotated Text*, 132); a second correction is Melville's reference to John Ledyard, an American adventurer who explored Africa and the Pacific with Captain Cook, which is replaced by the Scottish traveler in Africa Mungo Park; 128, 210 n. 30.

72 Thomas, *Cross-Examinations*, 108.

73 Lane, “Life and Times”; Franklin, “Past, Present, and Future Seemed,” 273; a fact Dampier conceals in his *Narrative*.

74 Farrier, *Unsettled Narratives*, 2. Melville was also familiar with earlier Spanish accounts of Pacific exploration such as Alvaro de Mendana's, which he references in *White-Jacket* (Brins 21).

75 Melville, “Encantadas or Enchanted Isles,” 257–258.

76 Jonik, “Melville's ‘Permanent Riotocracy’,” 248; Deleuze, “Bartleby.”

77 Jonik, “Melville's ‘Permanent Riotocracy’,” 230.

78 See “The Advocate,” ch. 24 of *Moby-Dick*; Jonik, “Melville’s ‘Permanent Riotocracy’,” 236; Wilson, *Reimagining the American Pacific*, 35; Eperjesi, *Imperialist Imaginary*, 29.

79 Melville, “Encantadas or Enchanted Isles,” 255.

80 Ibid., 261.

81 Kaplan, “Transnational Melville,” 52.

82 Nnolim, *Study in Meaning of Name Symbolism*, 18.

83 Emery, “Manifest Destiny,” 103.

84 On filibustering and US expansion in the period’s literature, see. Breinig, “Invasive Methods.”

85 Franklin, “Past, Present, and Future Seemed,” 238; see also Zagarell, “Reenvisioning America,” 141.

86 Mackenthun, *Fictions of the Black Atlantic*, 113, quoting Jones; also Franklin, “Past, Present, and Future Seemed,” 237; Beebee, “Carl Schmitt's Myth,” 128.

87 Sundquist, “New World Slavery,” 136.

88 Sale, *Slumbering Volcano*, 161. The tale includes a stereotype of African behavior as barbaric and brutal not to be found in Delano's source text. Goddard notes that Babo's name, too, alludes to historical pirates, namely the Barbary “pirates” and the conflict between North African states and western powers; “Of Mimicry and Masks,” 229.

89 Gillman and Gruesz, “Worlding America,” 238. Though he can only speak through the body, as Zagarell notes (“Reenvisioning America,” 136), Babo's final image is that of “an almost disembodied brain” (Karcher, *Shadow Over the Promised Land*, 130), perhaps the tale's most significant critique of racist assumptions about people of African descent.

90 Spanos, *American Calling*, 110.

91 Franklin, “Past, Present, and Future Seemed,” 237.

92 Stuckey, *African Culture*, 55.

93 Melville, *Moby-Dick*, 1199; Birns, “‘Oriental Archipelagoes’,” 13.

94 Sale, *Slumbering Volcano*, 158.

95 Haegert, “Voicing Slavery Through Silence,” 34.

96 Ibid., 177.

97 Levine, *Conspiracy and Romance*, 220.

98 Blum, “Atlantic Trade,” 120.

99 Ibid., 122; see also Mackenthun, *Fictions of the Black Atlantic*, ch. 5.

100 Buell, “American Decolonization,” 116.

101 Heide, “Inter-American Relations,” 37.

102 Ibid., 43.

103 Gillman and Gruesz, “Worlding America,” 231. Piracy is associated with early modern Europe earlier in “The Bell-Tower,” which examines the debauchery of the slave society of sixteenth-century Venice (Richardson, *Interpretation With Annotated Text*, 88) and connects the topic of slavery to its origins in European maritime states. Linked by Shakespeare’s *Othello* and its rebellious African protagonist in Venice, the diptych formed by the two tales presents a comment on the globalization of slavery (and piracy, for that matter, as the history of Venice is itself deeply entangled with piratical practices). Karcher views “The Bell-Tower” as more radical than “Benito Cereno,” confronting the reader “directly with a slave society's naked contempt for human values” (*Shadow*, 147). Venice is also invoked as another center of corruption in “The Town-Ho's Story,” ch. 54 of *Moby-Dick*.

104 Mackenthun, *Fictions of the Black Atlantic*, 122.

105 Levine, *Conspiracy and Romance*, 224.

106 Sundquist, “New World Slavery,” 107.

107 Strong, “‘Follow Your Leader’,” 284 (emphasis in the original).

108 Huang, *Transpacific Imaginations*, 8.

109 Ibid., 9.

110 James, *Mariners, Renegades and Castaways*, 133–134.

111 See also Franklin, “Past, Present, and Future Seemed.”

112 Sundquist, “New World Slavery,” 94.

113 Strong, “Follow Your Leader,” 288–289.

114 Balfour, “What Babo Saw,” 262 (emphasis in the original).

115 Schnackertz, “Wahrnehmungsperspektiven,” 179.

116 Strong, “Follow Your Leader,” 289.

117 Olson, *Call Me Ishmael*, 113.

118 Phillips, “Mapping Imagination,” 131.

119 Ibid., 133. Against this interpretation, John Carlos Rowe has read *Typee* as an anticipation of Melville's social criticism in his later work in that it refuses to represent the colonial experience as a “simple or singular narrative”; *Literary Culture*, 95.

120 Horne, *White Pacific.*

121 Huang, *Transpacific Imaginations*, 2.

122 Ibid., 3.

123 Wilson, *Reimagining the American Pacific*, 37.

124 Gniadek, “Americans Abroad,” 323.

125 Ibid., 326.
